# Normothermic *ex vivo* Heart Perfusion Combined With Melatonin Enhances Myocardial Protection in Rat Donation After Circulatory Death Hearts *via* Inhibiting NLRP3 Inflammasome-Mediated Pyroptosis

**DOI:** 10.3389/fcell.2021.733183

**Published:** 2021-08-31

**Authors:** Jun Lu, Liwei Xu, Zifeng Zeng, Chuqing Xue, Jiale Li, Xiong Chen, Pengyu Zhou, Shaoyan Lin, Yuhui Liao, Xianjin Du, Ronghua Yang, Shaoyi Zheng

**Affiliations:** ^1^Department of Cardiovascular Surgery, Nanfang Hospital, Southern Medical University, Guangzhou, China; ^2^Molecular Diagnosis and Treatment Center for Infectious Diseases, Dermatology Hospital, Southern Medical University, Guangzhou, China; ^3^Department of Critical Care Medicine, Renmin Hospital of Wuhan University, Wuhan, China; ^4^Department of Burn Surgery, The First People’s Hospital of Foshan, Foshan, China

**Keywords:** donation after circulatory death, normothermic *ex vivo* heart perfusion, melatonin, heart preservation, ischemia/reperfusion injury, pyroptosis

## Abstract

**Objective:**

The adoption of hearts from donation after circulatory death (DCD) is a promising approach for the shortage of suitable organs in heart transplantation. However, DCD hearts suffer from serious ischemia/reperfusion injury (IRI). Recent studies demonstrate that nucleotide-binding oligomerization domain-like receptor family pyrin domain-containing 3 (NLRP3) inflammasome-mediated pyroptosis is a novel target to ameliorate myocardial IRI. Melatonin is shown to inhibit NLRP3 inflammasome-mediated pyroptosis. Therefore, this study is designed to verify the hypothesis that melatonin can protect the heart graft preserved with *ex vivo* heart perfusion (EVHP) against myocardial IRI *via* inhibiting NLRP3 inflammasome-mediated pyroptosis in a rat model of DCD.

**Methods:**

Donor-heart rats were randomly divided into three groups: (1) Control group: non-DCD hearts were harvested from heart-beating rats and immediately preserved with allogenic blood-based perfusate at constant flow for 105 min in the normothermic EVHP system; (2) DCD-vehicle group; and (3) DCD-melatonin group: rats were subjected to the DCD procedure with 25 min of warm ischemia injury and preserved by the normothermic EVHP system for 105 min. Melatonin (200 μmol/L) or vehicle was perfused in the cardioplegia and throughout the whole EVHP period. Cardiac functional assessment was performed every 30 min during EVHP. The level of oxidative stress, inflammatory response, apoptosis, and NLRP3 inflammasome-mediated pyroptosis of heart grafts submitted to EVHP were evaluated.

**Results:**

Twenty five-minute warm ischemia injury resulted in a significant decrease in the developed pressure (DP), d*P*/d*t*_*max*_, and d*P*/d*t*_*min*_ of left ventricular of the DCD hearts, while the treatment with melatonin significantly increased the DP, d*P*/d*t*_*max*_ of the left ventricular of DCD hearts compared with DCD-vehicle group. Furthermore, warm ischemia injury led to a significant increase in the level of oxidative stress, inflammatory response, apoptosis, and NLRP3 inflammasome-mediated pyroptosis in the hearts preserved with EVHP. However, melatonin added in the cardioplegia and throughout the EVHP period significantly attenuated the level of oxidative stress, inflammatory response, apoptosis, and NLRP3 inflammasome-mediated pyroptosis compared with DCD-vehicle group.

**Conclusion:**

EVHP combined with melatonin post-conditioning attenuates myocardial IRI in DCD hearts by inhibiting NLRP3 inflammasome-mediated pyroptosis, which might expand the donor pool by the adoption of transplantable DCD hearts.

## Introduction

Heart transplantation remains the gold standard for the treatment of patients with refractory heart failure (Burchill and Ross, 2012). Unfortunately, despite the ever-increasing population of heart failure patients, the development of heart transplantation has been limited by the shortage of suitable donor hearts (Christie et al., 2012). Recently, the adoption of hearts from donation after circulatory death (DCD) has been considered as a promising approach to expanding the donor pool (Smith et al., 2019). DCD heart transplantation has the potential to significantly increase transplant activity by 30% in the United States (Jawitz et al., 2020) and 48% in the United Kingdom (Messer et al., 2020), and will result in a substantial decrease in waiting list mortality. Furthermore, DCD heart transplantation provides comparable 30-day or 1-year postoperative survival in comparison with traditional donation after brain death (DBD) heart transplantation (Messer et al., 2017, 2020). Nevertheless, DCD hearts suffer from more serious ischemia/reperfusion injury (IRI) due to an obligatory warm ischemia time (from when the systolic blood pressure is lower than 50 mmHg after the withdrawal of life-sustaining therapy to reperfusion or cardioplegia) (Dhital et al., 2017; Niederberger et al., 2019).

Recent studies have demonstrated that nucleotide-binding oligomerization domain-like receptor pyrin domain-containing 3 (NLRP3) inflammasome-mediated pyroptosis can play an indispensable role in the myocardial IRI (Dai et al., 2020; Wei et al., 2020; Xu L.J. et al., 2020). Pyroptosis is a recently identified type of programmed cell death and is characterized as a NLRP3-caspase-1-dependent response during myocardial IRI (Wu et al., 2020). NLRP3, one of the best characterized intracellular pattern recognition receptors, can be activated by the generation of reactive oxygen species (ROS) shortly after reperfusion. NLRP3 can bind to apoptosis-associated specklike protein containing a caspase recruitment domain (ASC) and finally form an NLRP3 complex (Geldhoff et al., 2013; Cero et al., 2015; Martínez et al., 2015), which will lead to the activation of caspase-1 and the production of interleukin-1β (IL-1β) and IL-18, and the recruitment of pro-inflammatory response in the progression of myocardial IRI (Bracey et al., 2015). Moreover, active caspase-1 can result in the cleavage of gasdermin D (GSDMD), a member of the gasdermin family, thereby inducing pyroptosis (Wang et al., 2020). Therefore, the interference of NLRP3 inflammasome-mediated pyroptosis might be a promising target for alleviating myocardial IRI of DCD hearts.

Static cold storage has been regarded as a traditional and reliable heart preservation strategy, which is simple, cheap, and able to preserve standard-criteria donor hearts for 4–6 h with acceptable post-transplant outcomes (Lund et al., 2017). However, static cold storage may not be the optimal preservation method for DCD hearts since the energy-depleted DCD hearts can poorly tolerate additional cold ischemia (Iyer et al., 2015). Recently, normothermic *ex vivo* heart perfusion (EVHP) is introduced as a promising preservation strategy for DCD hearts. Normothermic EVHP can perfuse donated hearts with warm, oxygenated, and nutrient-enriched blood-based perfusate in a semi-physiologic state during the preservation period (Zhou et al., 2020). Therefore, compared with static cold storage, normothermic EVHP can alleviate myocardial IRI for DCD hearts, prolong preservation time, allow for a real-time assessment of contractile function for DCD hearts, and offer a unique platform to repair DCD hearts *via* directly delivering post-conditioning agents into machine perfusion circuit (Beuth et al., 2019; Xu J. et al., 2020).

Melatonin (N-acetyl-5-methoxytryptamine), which is mainly synthesized by the pineal gland in mammals, is regarded as an anti-oxidant (Manchester et al., 2015), anti-inflammatory (Agil et al., 2013), and anti-apoptotic (Li et al., 2014) molecule. Notably, the pharmacological actions of melatonin are closely related to the inhibition of NLRP3 inflammasome-mediated pyroptosis. One recent study conducted by Su et al. (2021) showed that melatonin alleviated lipopolysaccharide-induced myocardial injury by inhibiting NLRP3 inflammasome-mediated pyroptosis. The administration of melatonin prevented endothelial cell pyroptosis *via* MEG3/miR-223/NLRP3 axis in atherosclerosis (Zhang et al., 2018). What’s more, melatonin can exert neuroprotective effects by inhibiting neuronal pyroptosis in the streptozotocin-induced diabetic mice (Che et al., 2020). However, it remains unclear whether normothermic EVHP combined with melatonin can ameliorate myocardial IRI for DCD hearts *via* inhibiting NLRP3 inflammasome-mediated pyroptosis. Additionally, until now, no study investigated the cardioprotective effect of the normothermic EVHP combined with melatonin post-conditioning in the preservation of donor hearts.

Therefore, in the present study, we explored the cardioprotective potential of melatonin post-conditioning during our well-established normothermic EVHP protocol in a rat model of DCD. We hypothesized that the combination of normothermic EVHP and melatonin post-conditioning could be a novel and promising donor heart preservation strategy, which could ameliorate myocardial IRI and improve cardiac function of DCD hearts, thereby increasing the number of transplantable hearts in heart transplantation.

## Materials and Methods

### Animals

Male Sprague-Dawley rats (Charles River Laboratories, Beijing, China) used in this study received care in compliance with the Guide for the Care and Use of Laboratory Animals (National Institutes of Health Publication No. 85-23, revised 1996). All animal experiments were reviewed and approved by the Ethical Committee of the Laboratory Animal Research Center of Southern Medical University Nanfang Hospital. The rats were housed in temperature-controlled (22 ± 2°C) rooms with a 12-h light-dark cycle, given food and sterilized water, and acclimatized for 1 week.

### Experiment Design

**FIGURE 1:**
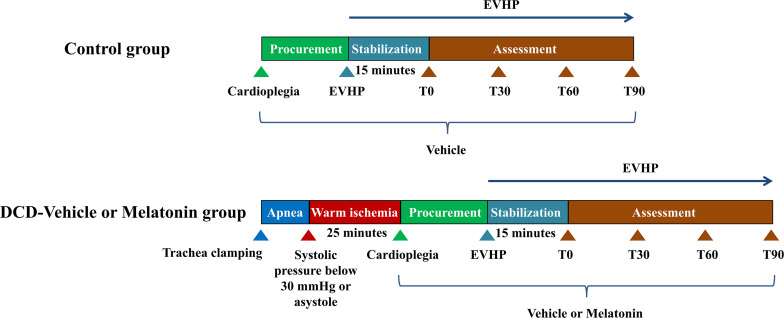
Study protocol of *ex vivo* heart perfusion combined with melatonin in the DCD heart preservation. DCD, donation after circulatory death; EVHP, *ex vivo* heart perfusion.

Twenty four male Sprague-Dawley rats (200–300 g; 8 to 12-week-old) were introduced as donor-heart rats. Another 24 male Sprague-Dawley rats (300–400 g; 12 to 15-week-old) were regarded as blood donors for blood-based perfusate of the normothermic EVHP system. All donor hearts were harvested and preserved by the normothermic EVHP system. Functional assessment of hearts was performed during the EVHP period. Heart tissue was collected at the end of perfusion for the evaluation of the level of oxidative stress, apoptosis, inflammation, and NLRP3 inflammasome-mediated pyroptosis (Figure 1).

Donor-heart rats were randomly divided into three groups: (1) control group: eight non-DCD hearts were harvested from heart-beating rats and immediately preserved by the normothermic EVHP system for 105 min. During harvesting the donor heart, the heart was perfused with 20 ml cold Custodiol (Dr. Franz Köhler, Chemie GmbH, Bensheim, Germany) cardioplegic solution containing 116 μl ethanal. Before initiating normothermic EVHP, 87 μl ethanal was added into 15 ml blood-based perfusate; (2) DCD-vehicle group; and (3) DCD-melatonin group: 8 rats were subjected to the DCD procedure with 25 min of warm ischemia injury and preserved by the normothermic EVHP system for 105 min. During harvesting the donor heart, the heart was perfused with 20 ml cold Custodiol cardioplegic solution containing 116 μl ethanal or melatonin (34.44 μmol/ml, dissolved in ethanol). Before initiating normothermic EVHP, 87 μl ethanal or melatonin (34.44 μmol/ml, dissolved in ethanol) was added into 15 ml blood-based perfusate to make the concentration of melatonin in the perfusate of 200 μM.

### Operative Procedure

#### Anesthesia

Sprague-Dawley rats were anesthetized with pentobarbital sodium (60 mg/kg, intraperitoneally). Pedal reflex was performed to determine adequate anesthetic depth before experiments. The rats were placed on a heating pad to maintain the body temperature.

#### Harvest of Blood From the Donor-Blood Rat

An 18 G, 2-inch I.V. catheter was cannulated into the abdominal artery. A 20 ml syringe containing 1,250 IU heparin was connected to the catheter to withdraw 9 ml blood. The blood-based perfusate consisted of 9 ml blood from the donor-blood rat, 6 ml modified Krebs–Henseleit solution (10 mM glucose, 117 mM NaCl, 4.5 mM KCl, 25 mM NaHCO_3_, 1.2 mM NaH_2_PO_4_, 2 mM CaCl_2_, 0.512 mM MgCl_2_), mannitol (25 g/L), methylprednisolone sodium succinate (500 mg/L; Pfizer; Belgium; Switzerland), and insulin (160 IU/L; Novo Nordisk; Denmark) (see supplementary material). The blood-based perfusate was slowly added into the perfusion circuit to be oxygenated for 15 min (PaO_2_ at 150–250 mmHg).

#### Procurement of Heart From the Donor-Heart Rat

The rat was intubated with a 16 G, 2-inch I.V. catheter after tracheotomy and mechanically ventilated. A 22 G, 1-inch I.V. catheter was cannulated into the right carotid artery for injection of heparin, real-time blood pressure monitoring, and delivery of Custodiol cardioplegic solution. The injection of heparin (2,000 IU/kg body weight) through the right carotid artery was performed to heparinize the donor-heart rat before the induction of circulatory death.

Rats in the DCD-vehicle and DCD-melatonin groups were subjected to the following DCD procedure. Briefly, the rat was extubated and the trachea was clamped by a mosquito forceps to induce circulatory death. Circulatory death could be declared when systolic pressure below 30 mmHg or asystole was observed (Kearns et al., 2017; Aceros et al., 2020). At the end of the 25-min warm ischemic time (WIT, equivalent to the hands-off period), we performed the sternotomy and clamped the aortic arch between the brachiocephalic trunk and left common carotid artery. After cutting the inferior vena cava and opening the left atrium, the DCD heart was immersed with ice and perfused with 20 ml cold Custodiol cardioplegic solution containing 116 μl ethanal (DCD-vehicle group) or melatonin (DCD-melatonin group) at a constant pressure *via* the right carotid artery. The aorta distal to the left subclavian artery and pulmonary artery were cut, respectively. Subsequently, the arrested heart was excised and cannulated with a homemade 14 G aortic catheter. The rats in the control group were treated similarly, but not submitted to 25-min WIT.

#### The Operation of EVHP

**FIGURE 2:**
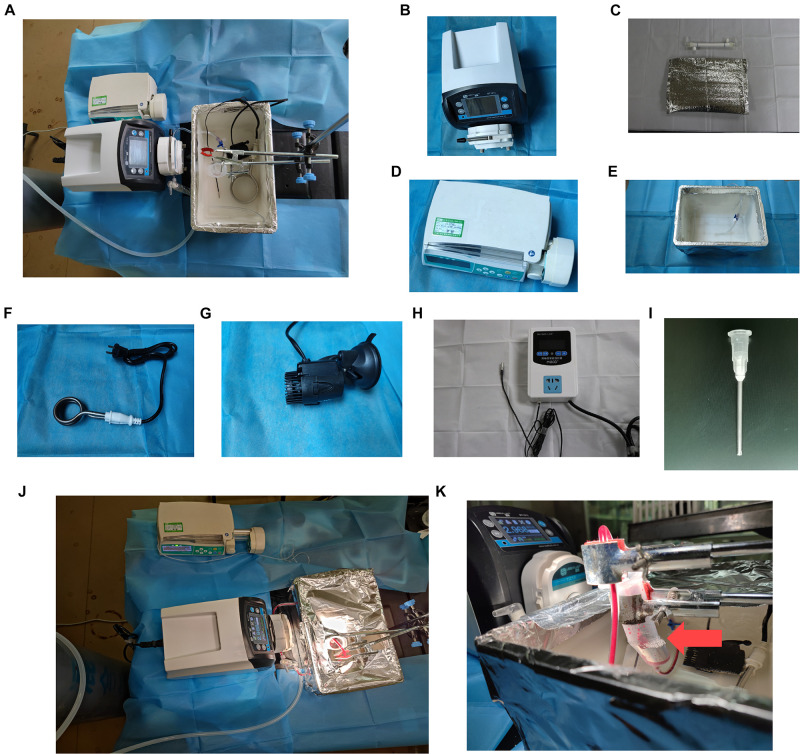
*Ex vivo* heart perfusion system. **(A)** Schematic figure of the EVHP system; **(B)** micro-peristaltic pump; **(C)** oxygenator; **(D)** infusion syringe pump; **(E)** water-bath box; **(F)** heater; **(G)** stirrer; **(H)** temperature-controlling switch; **(I)** aortic catheter; **(J)** working EVHP system; and **(K)** beating heart (red arrow). EVHP, *ex vivo* heart perfusion.

The normothermic ESHP system (Figure 2A) consisted of a micro-peristaltic pump (BT101L; Lead Fluid; China; Figure 2B), an oxygenator (Micro-1 Rat Oxygenator; Dongguan Kewei; China; Figure 2C), 16# Tygon tubing, a reservoir, an infusion syringe pump (Perfusor-space; B. Braun; Germany; Figure 2D) for the administration of epinephrine, and a home-made water-bath box (Figure 2E) containing water, heater (Figure 2F), stirrer (Figure 2G), temperature-controlling switch (Figure 2H), and aortic cannula (Figure 2I). The oxygenator was gassed with a humidified gas mixture containing 95% O_2_/5% CO_2_.

The donor heart was connected to the EVHP system *via* the aortic cannula (Figures 2J,K). The reservoir was partially immersed in the water of the water-bath box and the isolated heart was placed below the horizontal plane of the water. The membrane oxygenator was wrapped by a thermal insulation bag and the temperature of water in the box was set at 41–42 °C to maintain the temperature of the inflow and isolated heart at 35–37°C (Figure 2K). The normothermic EVHP started with the perfusion flow rate of 2 ml/min, and slowly reached the target perfusion flow rate (1 ml/min/100 g body weight) within 10 min. The administration of epinephrine (4.8 × 10^–5^mg/h/kg body weight) was performed *via* an infusion syringe pump throughout the perfusion after a 15-min stabilization period. During the normothermic EVHP, PaO_2_ was maintained at 150–250 mmHg and the temperature of the isolated heart at 35–37°C. 20 μl 50% glucose solution and 40 μl 5% sodium bicarbonate solution were added into the perfusate every 15 min.

#### Cardiac Functional Assessment During EVHP

The latex balloon connected to a pressure sensor was inserted into the left ventricle through the left atrium at the end of the 15-min stabilization period. The intraventricular pressure measurement of the donor heart was performed by slowly filling the balloon with 0.15 ml saline at the beginning of the assessment phase (T0) and then every 30 min (T30, T60, and T90). The cardiac functional parameters of the donor heart during EVHP included developed pressure (DP, systolic blood pressure minus diastolic blood pressure), heart rate (HR), d*P*/d*t*_*max*_ (maximum rate of rise of left ventricular pressure), and d*P*/d*t*_*min*_ (maximum rate of pressure decline).

#### Sample Collection

At the end of 105-min EVHP, three ventricular slices (1- to 2-mm thick) were collected serially along the long axis. The first piece of myocardial tissue from the apex was applied for western blotting, the second one for histologic and immunohistochemical analysis, the third one for analyzing superoxide dismutase (SOD) activity, and the level of malonaldehyde (MDA).

#### Oxidative Stress

The SOD activity and the level of MDA in the heart tissue of donor heart were determined by colorimetric 5,5′-dithio-bis-(2-nitrobenzoic acid)-based method and thiobarbituric acid reactive substance assay, respectively (A001-3-2 and A003-1-2, Nanjing Jiancheng Bioengineering Institute, China). The analysis was performed using an automatic microplate reader (CLARIOstar, BMG LAGTECH, Germany). The expression of 4-hydroxynonenal (HNE), an indicator of oxidative stress, was determined by immunohistochemical analysis as described below.

#### Inflammatory Response

The expression of interleukin-6 (IL-6), tumor necrosis factor-α (TNF-α), and nuclear factor kappa-B p65 (NF-κB p65) were measured by immunohistochemistry as described below.

#### Immunohistochemistry

Myocardial tissue slices were fixed in paraformaldehyde solution (4%), embedded in paraffin, and cut into 4-μm-thick sections. The immunoreactivity to IL-6 (1:500, Abcam, ab9324, United States), TNF-α (1:200, Abcam, ab109322, United States), NF-κB p65 (1:500, Proteintech, 66535, China), HNE (1:1,000, Abcam, ab46545, United States) was assessed. The antigen–antibody reaction was visualized by diaminobenzidine reaction. The fields from each slice were randomly selected and recorded under a conventional light microscope in a blinded manner. Image analysis was performed using Image-Pro Plus software (Media Cybernetics, United States). The evaluation was performed in four random and non-overlapping fields of the heart tissue, and the average value was calculated for each animal. The expression of IL-6, TNF-α, NF-κB p65, and HNE was determined by counting integrated optical density (IOD).

#### Apoptosis

Terminal deoxynucleotidyl transferase dUTP nick end labeling (TUNEL) staining was performed to detect DNA-strand breaks of the donor hearts as previously described (Zhou et al., 2021). The number of TUNEL-positive cells was counted under a fluorescence microscope and the frequency of apoptosis in the donor heart was expressed as the ratio of 4′,6-diamidino-2-phenylindole (DAPI)-TUNEL double-labeled nuclei to the total number of nuclei stained with DAPI.

#### Western Blotting

Myocardial protein expression of donor heart was assessed by western blotting as previously described (Korkmaz-Icöz et al., 2020). The expression of cleaved caspase-3 (1:1,000 dilution, 9664, Cell Signaling Technology (Shanghai) Biological Reagents Company Limited, China), NLRP3 [1:1,000 dilution, 15101, Cell Signaling Technology (Shanghai) Biological Reagents Company Limited, China], ASC [1:1,000 dilution, 67824, Cell Signaling Technology (Shanghai) Biological Reagents Company Limited, China], Caspase-1 (1:1,000 dilution, ab179515, Abcam, United States), IL-1β (1:1,000 dilution, ab9722, Abcam, United States), IL-18 (1:1,000 dilution, ab207323, Abcam, United States), and cleaved GSDMD [1:1,000 dilution, 10137, Cell Signaling Technology (Shanghai) Biological Reagents Company Limited, China] were evaluated.

#### Statistical Analysis

The results were expressed as mean ± standard error of the mean (SEM). GraphPad Prism 8.3 software (GraphPad Sofware, Inc., San Diego, CA, United States) was used to perform statistical analysis. Shapiro-Wilk test was performed to test the normality of data before statistical tests were applied. One-way ANOVA followed by Tukey’s *post hoc*-test was performed for multiple comparisons between three experimental groups. If the data failed the normality test, the non-parametric Kruskal–Wallis test followed by Dunn’s *post hoc*-test was used. Intraventricular pressure measurement recordings among control, DCD-vehicle, and DCD-melatonin groups were compared by two-way ANOVA according to different timepoints. A value of *p* < 0.05 was considered statistically significant.

## Results

### *Ex vivo* Heart Perfusion Combined With Melatonin Post-conditioning Protects Against Cardiac Function Impairment Caused by Warm Ischemia Injury in the DCD Hearts

**FIGURE 3:**
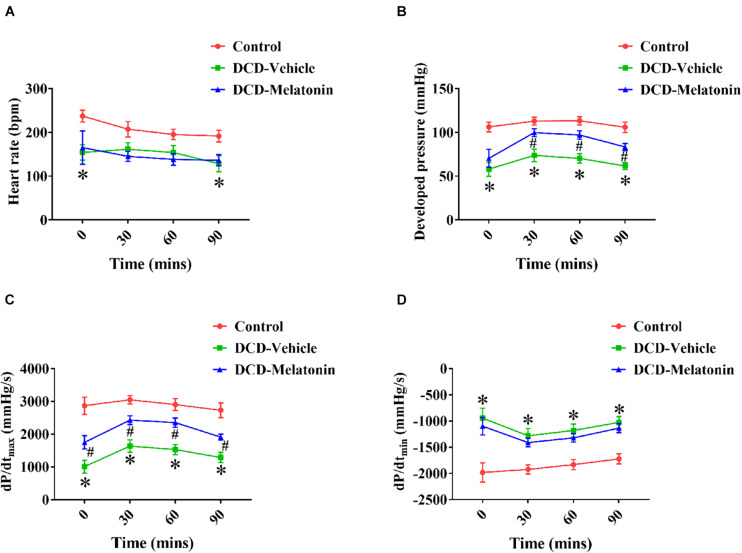
Cardiac functional assessment of the DCD heart during EVHP. **(A)** Heart rate; **(B)** developed pressure; **(C)** d*P*/d*t*_*max*_; and **(D)** d*P*/d*t*_*min*_. Data represent mean ± standard error of the mean. *n* = 8 for each group. DCD, donation after circulatory death; EVHP, *ex vivo* heart perfusion; d*P*/d*t*_*max*_, maximum rate of rise of left ventricular pressure; d*P*/d*t*_*min*_, maximum rate of pressure decline. ^∗^*p* < 0.05 vs. control; ^#^*p* < 0.05 vs. DCD-vehicle.

A WIT of 25 min results in a significant decrease in HR (T0 and T9, Figure 3A), DP (from T0 to T90, Figure 3B), d*P*/d*t*_*max*_ (from T0 to T90, Figure 3C), and d*P*/d*t*_*min*_ (from T0 to T90, Figure 3D) of DCD hearts preserved with EVHP in the DCD-vehicle group compared with the control group. Furthermore, melatonin post-conditioning leads to a significant increase in DP (from T30 to T90, Figure 3B) and d*P*/d*t*_*max*_ (from T0 to T90, Figure 3C) of DCD hearts preserved with EVHP in DCD-melatonin group compared with DCD-vehicle group.

### *Ex vivo* Heart Perfusion Combined With Melatonin Post-conditioning Reduces Oxidative Stress in the DCD Hearts

**FIGURE 4:**
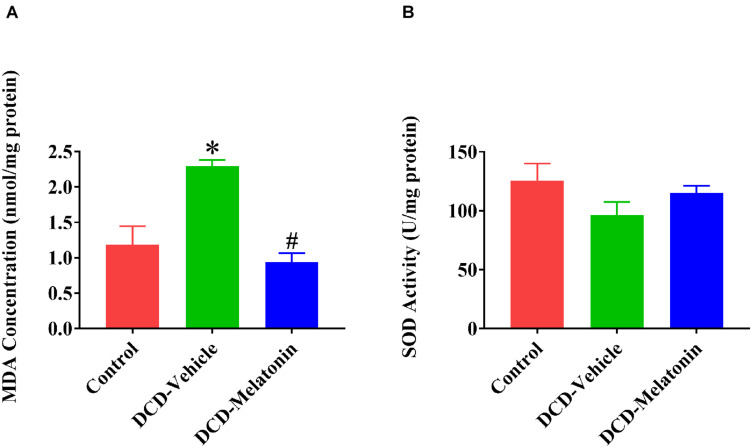
Tissue content of MDA and SOD activity in the DCD heart preserved with EVHP. **(A)** Tissue content of MDA; and **(B)** SOD activity. Data represent mean ± standard error of the mean. *n* = 5 for each group. DCD, donation after circulatory death; EVHP, *ex vivo* heart perfusion; MDA, malonaldehyde; and SOD, superoxide dismutase. ^∗^*p* < 0.05 vs. control; ^#^*p* < 0.05 vs. DCD-vehicle.

**FIGURE 5:**
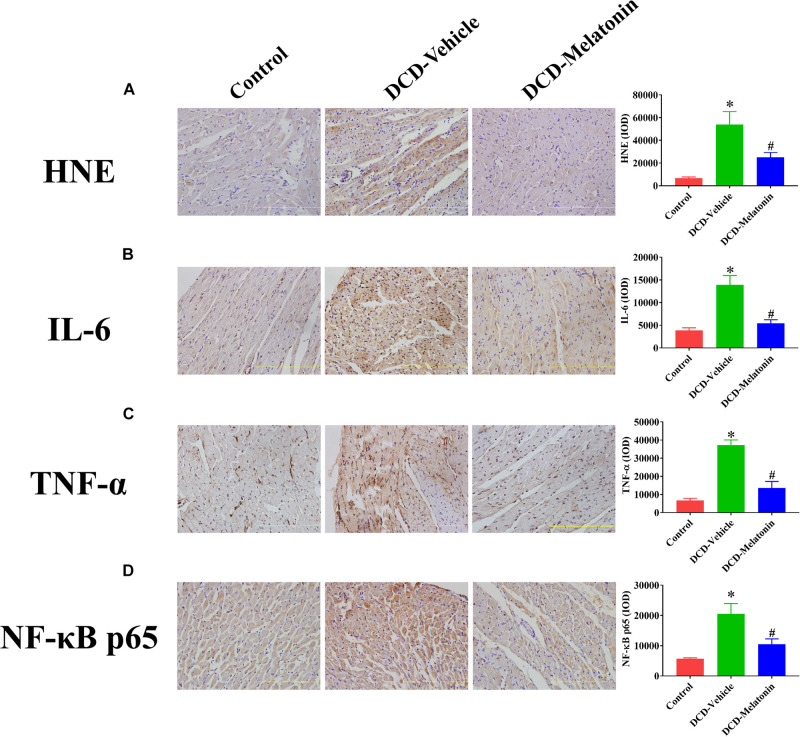
The expression of HNE, IL-6, TNF-α, and NF-κB p65 in the DCD heart preserved with EVHP. Representative photomicrographs and quantitative analysis of immunohistochemistry for **(A)** HNE, **(B)** IL-6, **(C)** TNF-α, and **(D)** NF-κB p65 in the DCD heart. Data represent mean ± standard error of the mean. *n* = 6–8 for each group. DCD, donation after circulatory death; EVHP, *ex vivo* heart perfusion; HNE, 4-hydroxynonenal; IL-6, interleukin-6; IOD, integrated optical density; TNF-α, tumor necrosis factor-α; NF-κB, nuclear factor kappa-B. ^∗^*p* < 0.05 vs. Control; ^#^*p* < 0.05 vs. DCD-vehicle.

Malonaldehyde is introduced as a reliable marker of oxidative stress since it is the end product of major reactions which can lead to significant oxidation of polyunsaturated fatty acids in cellular membranes (Reiter et al., 2009). A WIT of 25 min can significantly increase the level of MDA in the DCD hearts preserved with EVHP in the DCD-vehicle group compared with the control group. Treatment with melatonin at 200 μmol/L during cardioplegia and through the EVHP period can significantly reduce the level of MDA in the DCD hearts in the DCD-melatonin group compared with the DCD-vehicle group (Figure 4A). SOD is an enzyme that can catalyze the dismutation of the superoxide (O_2_^–^) radical into ordinary molecular oxygen (O_2_), thereby exhibiting a great anti-oxidant effect (Hayyan et al., 2016). As shown in Figure 4B, the SOD activity of the heart graft in the control group and DCD-melatonin group is higher than that in the DCD-vehicle group without reaching a significant difference. The expression of HNE, an indicator of oxidative stress, among three groups was determined by immunohistochemical analysis. A WIT of 25 min leads to a significant increase in the expression of HNE in the DCD-vehicle group compared with the control group, whereas the treatment with melatonin can significantly down-regulate the expression of HNE in the DCD hearts preserved with EVHP (Figure 5A).

Collectively, 25-min warm ischemia injury increases the level of oxidative stress in the heart graft preserved with EVHP, while the addition with melatonin at 200 μmol/L during cardioplegia and EVHP period attenuates the level of oxidative stress in the DCD heart.

### *Ex vivo* Heart Perfusion Combined With Melatonin Post-conditioning Attenuates Inflammation in the DCD Hearts

The expression of IL-6, TNF-α, and NF-κB in heart tissue was measured by immunohistochemistry to evaluate the inflammatory response in the myocardium. As shown in Figure 5, a WIT of 25 min can significantly up-regulate the levels of IL-6 (Figure 5B), TNF-α (Figure 5C), and NF-κB (Figure 5D) in the DCD heart submitted to EVHP, while the treatment of melatonin can lead to a significant decrease in the expression of IL-6, TNF-α, and NF-κB in the DCD heart.

### *Ex vivo* Heart Perfusion Combined With Melatonin Post-conditioning Ameliorates Apoptosis in the DCD Hearts

**FIGURE 6:**
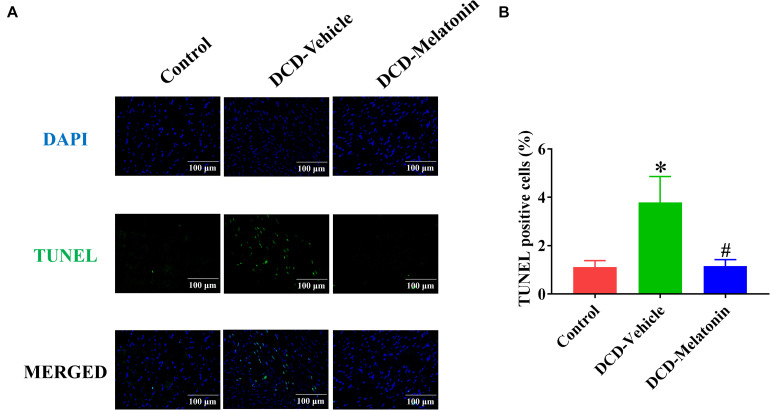
Apoptosis evaluation of the DCD heart submitted to EVHP. **(A)** Representative photomicrographs of myocardial tissue stained with DAPI (blue), nuclei with fragmented DNA shown by TUNEL staining, and merged image (magnification of 40; scale length: 100 μm); and **(B)** quantification of TUNEL-positive cells (as a percentage). Data represent mean ± standard error of the mean. *n* = 8 for each group. DAPI, 4′,6-diamino-2-phenylindole (DAPI, blue); DCD, donation after circulatory death; EVHP, *ex vivo* heart perfusion; TUNEL, terminal deoxynucleotidyl transferase-mediated dUTP nick end-labeling. ^∗^*p* < 0.05 vs. control; ^#^*p* < 0.05 vs. DCD-vehicle.

**FIGURE 7:**
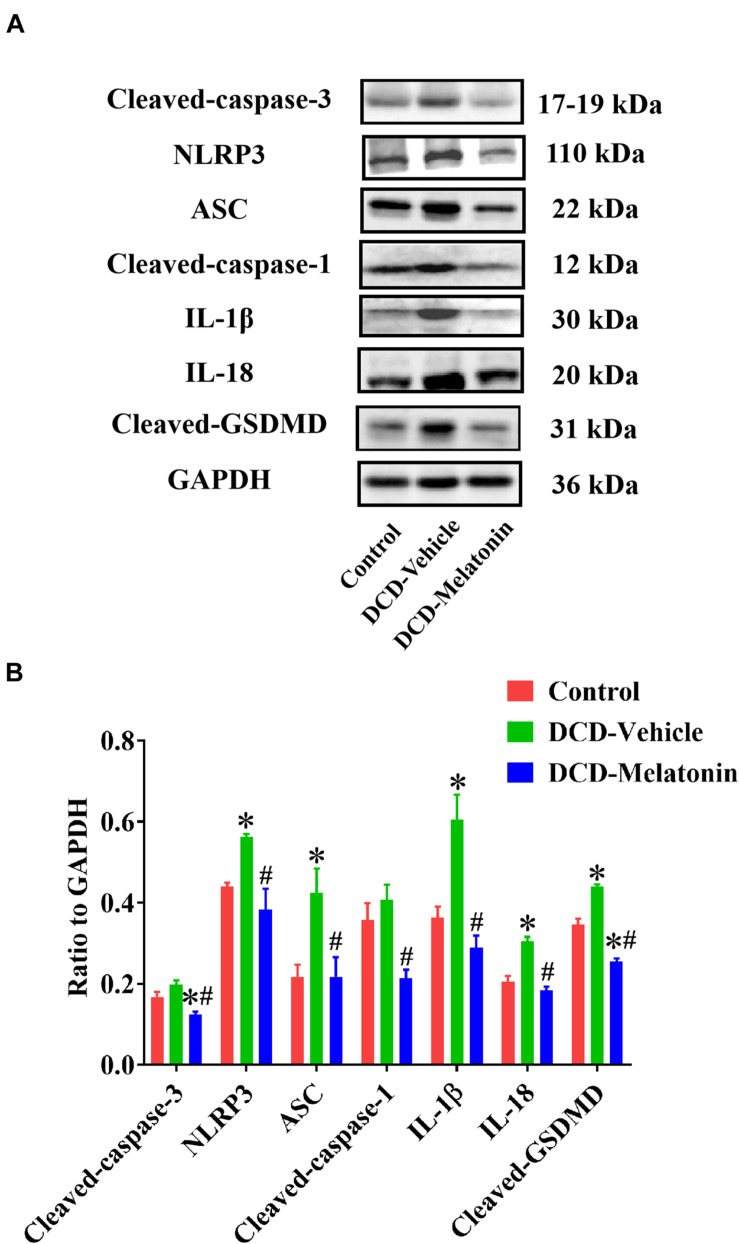
Representative Western blotting and quantitative analysis of the effect of melatonin treatment on apoptosis and NLRP3 inflammasome-mediated pyroptosis in the hearts preserved with EVHP. **(A)** Representative protein band densities of cleaved caspase-3, NLRP3, ASC, cleaved caspase-1, IL-1β, IL-18, and cleaved GSDMD; and **(B)** quantitative analysis of protein expression in the DCD hearts. Data represent mean ± standard error of the mean. *n* = 4 for each group. DCD, donation after circulatory death; EVHP, *ex vivo* heart perfusion. ^∗^*p* < 0.05 vs. Control; ^#^*p* < 0.05 vs. DCD-vehicle.

After normothermic EVHP, a significantly increased number of TUNEL-positive nuclei in the heart graft can be observed in the DCD-vehicle group compared with the control group. Besides, treatment with melatonin significantly decreases DNA strand breaks and the expression of cleaved caspase-3 in the myocardium of the DCD hearts preserved with EVHP compared with DCD-vehicle group (Figures 6, 7).

### *Ex vivo* Heart Perfusion Combined With Melatonin Post-conditioning Inhibits NLRP3 Inflammasome-Mediated Pyroptosis in the DCD Hearts

To investigate whether NLRP3 inflammasome-mediated pyroptosis is involved in myocardial IRI for the DCD heart, and the relationship between NLRP3 inflammasome-mediated pyroptosis and melatonin treatment in the DCD heart, the expression of NLRP3, ASC, cleaved caspase-1, IL-1β, IL-18, and cleaved GSDMD is evaluated by western blotting analysis.

As shown in Figure 7, the protein level of NLRP3, ASC, IL-1β, IL-18, and cleaved GSDMD in the heart submitted to EVHP is significantly higher in the DCD-vehicle group compared with the control group, while the treatment of melatonin can attenuate NLRP3 inflammasome-mediated pyroptosis (as evidenced by the significantly increased expression of NLRP3, ASC, cleaved caspase-1, IL-1β, IL-18, and cleaved GSDMD) in the DCD heart preserved with EVHP.

## Discussion

Currently, heart transplantation with DCD hearts has received enormous attention over the past 15 years (Osaki et al., 2010; Manara et al., 2012) and is emerging as a promising strategy to expand the donor pool (Longnus et al., 2014). Nevertheless, the use of DCD hearts has been hampered by the severe IRI due to the inherent warm ischemia injury (Citerio et al., 2016; Stehlik et al., 2018; Smith et al., 2019). Normothermic EVHP is becoming a novel and ground-breaking strategy for DCD heart preservation in the clinical setting. Unlike static cold storage, the traditional organ preservation method, normothermic EVHP can preserve the donated heart in a perfused, semi-physiologic state (Van Raemdonck et al., 2013), thereby reducing myocardial IRI, allowing for a thorough evaluation for the viability of donor heart (Xu J. et al., 2020), and more excitingly, facilitating organ reconditioning during perfusion with pharmacological, gene, and stem cell therapy (Weissenbacher and Vrakas, 2019). Therefore, the combination of normothermic EVHP with some novel post-conditioning pharmacological agents could hopefully convert DCD hearts into transplantable donor hearts.

**FIGURE 8:**
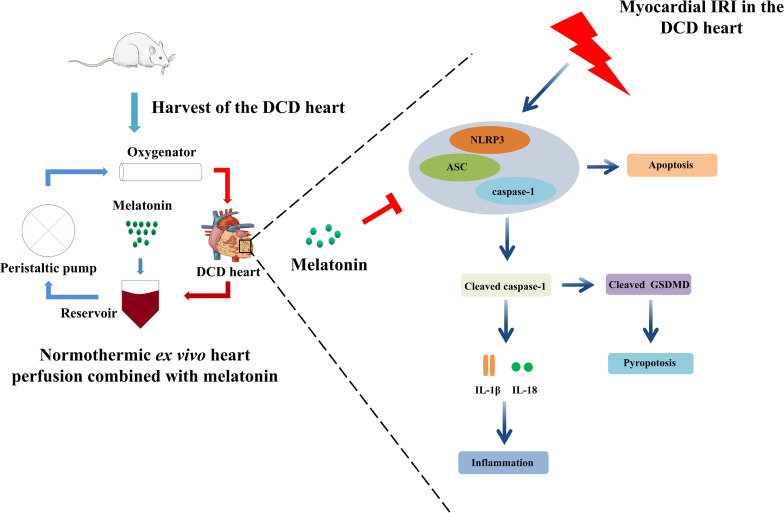
*Ex vivo* heart perfusion combined with melatonin post-conditioning exerts a profound cardioprotective effect against myocardial IRI in the DCD heart, and this protection appears to be due to the inhibition of NLRP3 inflammasome-mediated pyroptosis. IRI, ischemia/reperfusion injury; DCD, donation after circulatory death.

Previous studies proved the cardioprotective effect of melatonin, an important circadian molecule, which was either added into preservation solution in form of cold static storage (Rudd and Dobson, 2011) or administered for the donors (Lan et al., 2019) or recipients (Jung et al., 2004) in the heart transplantation. However, whether normothermic EVHP with the addition of melatonin can become a novel preservation strategy for the DCD heart remains unknown. The current study is the first time to demonstrate that melatonin has potent cardioprotective effects for the DCD heart when used throughout cardioplegia and EVHP period. Our results indicate that 25-min warm ischemia injury results in a significant decrease in cardiac functional parameters, and an increase in the level of oxidative stress, inflammatory response, apoptosis, and NLRP3 inflammasome-mediated pyroptosis of DCD hearts. However, the treatment with melatonin leads to a significant increase in cardiac functional parameters and attenuates myocardial IRI (as evidenced by the level of oxidative stress, inflammatory response, and apoptosis), and NLRP3 inflammasome-mediated pyroptosis of DCD hearts submitted to our well-established EVHP protocol (Figure 8).

Recently, an increasing number of studies have demonstrated that NLRP3 inflammasome-mediated pyroptosis is emerging as a novel target to attenuate myocardial IRI (Ding et al., 2020; Nie et al., 2021). Inhibiting microRNA-29a can alleviate myocardial IRI in mice by targeting SIRT1 and suppressing oxidative stress and the NLRP3-mediated pyroptosis pathway (Ding et al., 2020). Furthermore, hydrogen gas inhalation can attenuate myocardial IRI in rats by the inhibition of oxidative stress and NLRP3-mediated pyroptosis (Nie et al., 2021). Notably, Quader et al. (2020) reported that the DCD procedure resulted in the activation of the NLRP3 inflammasome, which contributed to myocardial damage and dysfunction, while NLRP3 inflammasome inhibition ameliorated myocardial warm ischemia injury and improved DCD heart function. In line with the above findings, our study shows 25-min warm ischemia in the DCD hearts impairs the cardiac function of hearts during 105-min EVHP and leads to a significant increase in the level of oxidative stress, inflammatory response, apoptosis, and NLRP3 inflammasome-mediated pyroptosis. Emerging studies have demonstrated that melatonin can effectively inhibit NLRP3 inflammasome-mediated pyroptosis. In agreement with these researches, the present study shows the mechanism of melatonin’s cardioprotection against myocardial IRI in DCD hearts preserved with EVHP is partly due to the inhibition of NLRP3 inflammasome-mediated pyroptosis.

In our study, no difference was found in left ventricular relaxation, as evidenced by d*P*/d*t*_*min*_, between DCD-melatonin and DCD-vehicle groups. Nevertheless, this may be due to the optimized protocol of DCD heart procurement and normothermic EVHP which can ensure cardioprotection from myocardial IRI. Besides, the current normothermic EVHP is limited to 105 min and a longer duration of reperfusion may be necessary to present better cardiac functional recovery in the melatonin-treated DCD hearts.

Apoptosis, another form of programmed cell death, can be characterized as cell shrinkage, nuclear fragmentation, and chromosomal DNA fragmentation. Furthermore, the inhibition of NLRP3 inflammasome can suppress the frequency of cell apoptosis (Wu et al., 2014; Li et al., 2015; Zhao et al., 2015). The current study reveals that the activation of NLRP3 inflammasome-mediated pyroptosis and increased apoptotic rate can contribute to myocardial IRI in the DCD heart, which can be inhibited by the treatment of melatonin. Therefore, we infer that normothermic EVHP combined with melatonin post-conditioning may attenuate myocardial apoptosis *via* inhibiting NLRP3 inflammasome-mediated pyroptosis.

There are several limitations to our present studies. Firstly, the duration of the current normothermic EVHP is limited to 105 min and should be prolonged in future studies to clearly present better cardiac functional recovery in the melatonin-treated DCD hearts. Secondly, a heart transplantation model should be introduced in the future to demonstrate the cardioprotective effect of normothermic EVHP combined with melatonin on the post-transplant donor heart. Thirdly, the concentration of melatonin in the cardioplegic solution and perfusate may not be optimal although previous studies have demonstrated that melatonin, given either acutely or chronically at pharmacological doses, has virtually no toxicity (Jahnke et al., 1999; Seabra et al., 2000). Therefore, the dose-effect relationship of melatonin in the DCD hearts preserved with EVHP should be investigated in the future. In parallel, future research is necessary to confirm the cardioprotective effect of normothermic EVHP combined with melatonin in a porcine model to obtain more robust evidence for an efficient clinical translation. Finally, the precise mechanism for inhibition of the NLRP3 inflammasome-mediated pyroptosis by melatonin needs to be further studied.

## Conclusion

Taken together, the present study reveals that normothermic EVHP combined with melatonin can attenuate myocardial IRI in the DCD hearts *via* inhibiting NLRP3 inflammasome-mediated pyroptosis. Therefore, the combination of normothermic EVHP and melatonin may facilitate the expansion of the DCD protocol for donor hearts by allowing the adoption of marginal organs, thereby improving the donor pool availability.

## Data Availability Statement

The original contributions presented in the study are included in the article/supplementary material, further inquiries can be directed to the corresponding authors.

## Ethics Statement

The animal study was reviewed and approved by the Ethical Committee of the Animal Experimental Center of Southern Medical University Nanfang Hospital.

## Author Contributions

JuL, LX, ZZ, and CX performed the experiments, analyzed the data, and participated in the writing of the manuscript. JiL, XC, PZ, and SL conducted the experiments and collected the data. YL, XD, RY, and SZ designed the experiments and revised the manuscript. All authors read and approved the final manuscript.

## Conflict of Interest

The authors declare that the research was conducted in the absence of any commercial or financial relationships that could be construed as a potential conflict of interest.

## Publisher’s Note

All claims expressed in this article are solely those of the authors and do not necessarily represent those of their affiliated organizations, or those of the publisher, the editors and the reviewers. Any product that may be evaluated in this article, or claim that may be made by its manufacturer, is not guaranteed or endorsed by the publisher.
